# Response of Pumpkin to Different Concentrations and Forms of Selenium and Iodine, and their Combinations

**DOI:** 10.3390/plants9070899

**Published:** 2020-07-16

**Authors:** Aleksandra Golob, Ana Kroflič, Ana Jerše, Nina Kacjan Maršić, Helena Šircelj, Vekoslava Stibilj, Mateja Germ

**Affiliations:** 1 Biotechnical Faculty, University of Ljubljana, SI 1000 Ljubljana, Slovenia; nina.kacjan.marsic@bf.uni-lj.si (N.K.M.); helena.sircelj@bf.uni-lj.si (H.S.); mateja.germ@bf.uni-lj.si (M.G.); 2 Jožef Stefan International Postgraduate School, SI 1000 Ljubljana, Slovenia; ana.kroflic@gmail.com (A.K.); ana.jerse@gmail.com (A.J.); 3 Department of Environmental Sciences, Jožef Stefan Institute, SI 1000 Ljubljana, Slovenia

**Keywords:** iodine, selenium, biofortification, *Cucurbita pepo* L. ssp. *pepo*, pumpkins seeds, sprouts, yield

## Abstract

The elements selenium (Se) and iodine (I) are both crucial for the normal functioning of the thyroid. Biofortification with these elements is particularly feasible in areas where they show a deficit. Iodine and selenium can have positive effects on different plants when applied at the correct concentrations. The effects of their simultaneous addition on plant physiology and biochemistry, as well as on seed germination and sprout biomass, were studied in pumpkin (*Cucurbita pepo* L. ssp. *pepo*). To study the effect of Se and I on sprouts, sprouts were grown from seeds soaked in solutions of different forms of Se, I and their combination in the growth chamber experiment. In the field experiment, pumpkins plants were foliarly treated with the same concentrations and forms of Se and I. The combination of Se and I treatments enhanced the germination of the soaked seeds, with no significant differences between Se and I treatments for sprout mass. The yield of pumpkins and seed production were unaffected by Se and I foliar application. The anthocyanin levels and respiratory potential measured via the electron transport system’s activity showed different patterns according to treatments and plant parts (sprouts, leaves, seeds). The redistribution of Se and I from seeds to sprouts was significant. The accumulation of Se was higher in sprouts from the seeds treated with Se together with I, compared to sprouts from the seeds treated with Se alone. Interactions between Se and I were also noted in the seeds, which developed in the treated plants.

## 1. Introduction

Selenium (Se) and iodine (I) are essential trace elements for human health. Iodine is a component of the thyroid hormones thyroxine (T4; 3,5,3′,5′-tetraiodothyronine) and T3 (3,5,3′-triiodothyronine). These hormones are important for metabolism, protein synthesis and enzymatic activities. Selenium is known to be a cofactor of the enzyme iodothyronine deiodinase, which converts hormone T4 to the active T3 [1,2]. Epidemiological studies have shown that a low intake of Se can increase the risk of cardiovascular disease and cancers. Selenium deficiency can cause various health problems, such as Keshan and Kaschin–Beck diseases. 

Selenium is predominantly provided by cereals, meat and fish [3]. White and Broadley [4] stated that Se deficiency in humans prevails due to low Se concentrations and availability in soils, with consequentially low concentrations in crop plants. Agronomic biofortification is considered advantageous, especially as plants assimilate Se into the organic forms that are more available for humans (e.g., selenomethionine) [5] than direct Se supplementation, which can also suffer from the low bioavailability of the inorganic Se and possible accidental excessive Se intake by humans [6]. Foliar application is the most effective means of biofortification with Se [7]. As regards soil application, losses occur via soil adsorption, and chemically and microbiologically mediated conversion. Foliar spraying results in a high degree of uptake and assimilation by the plants. The efficiency of foliar spraying depends on ion absorption through the above-ground plant organs, and the Se mobility within the plant. Se ions can enter the inner tissue of the leaf, where high concentrations can damage cells. From there, Se ions are distributed among the other plant organs [8,9].

Iodine is involved in human metabolic growth, development and regulation, and a deficit can therefore result in a wide range of diseases. These are designated as I-deficiency diseases [4], and they represent one of the most common preventable human health problems. The degrees of accumulation and enrichment of I in various vegetables, and in various parts of any one vegetable, are different. Thus, the concentration of I in a plant is determined by the plant type and by the physiological activity of the plant [4]. The biofortification of certain plants with I might represent an alternative method to the iodisation of table salt, for introducing I into the human diet and preventing I deficiency and the related human disorders [10]. The effects of I on plants involve antioxidative actions and mineral nutrition [11,12,13,14]. Approximately two-thirds of the whole human population suffer from illnesses and health problems caused by insufficient supplies of Se and I in their daily diet [15]. 

Plant roots can take up Se as selenate (SeO_4_^2−^), selenite (SeO_3_^2−^) or organoselenium compounds, such as selenocysteine and selenomethionine. Selenite is readily converted into organoselenium compounds in the roots, while SeO_4_^2−^ is delivered to the xylem and transported to the shoots, where it is assimilated into organoselenium compounds and redistributed within the plant [4]. In most soils, I is present as I^−^, with IO_3_^−^ also present under strongly oxidising conditions. Although I is not readily mobile in the plant phloem, I concentrations in tubers, fruit and seeds can be increased by I fertilisation, to nutritionally significant concentrations [4,16]. Smoleń et al. [15] indicated a synergistic interaction between IO_3_^−^ and the SeO_4_^2−^ absorption by the leaves of lettuce during foliar applications of these two compounds. 

Although it is widely recognised that Se and I are both needed for the proper functioning of the thyroid in humans and animals, interactions between Se and I in plant uptake processes have just recently been reported in the literature [15,16,17,18,19,20,21,22]. In addition, there are few data available on the interactions between these two elements in terms of their negative or positive effects on the physiological and biochemical characteristics of plants. Golob et al. [16] reported increased contents of photosynthetic pigments in the leaves of plants sprayed with a combination of Se and I in water solution. 

The pumpkin (*Cucurbita pepo* L. ssp. *pepo*) is an annual herbaceous plant that belongs to the Cucurbitaceae family. It produces large fruits with oily seeds [23]. It has been shown that pumpkin seeds from Se-enriched plants are useful in improving the nutritional status of Se in humans [24]. Pumpkin crops are traditional in central Europe, because of their high nutritional value and particular culinary attraction. The seeds are known for their high content of sterols, vitamins B and E, and the minerals Ca, K, P, Mg, Fe and Zn. In northeastern Slovenia (Prlekija and Prekmurje regions), in southeastern Austria and in southwestern Hungary, pumpkin seeds are used for the production of edible oil. The oil content of pumpkin seeds varies, and is genetically determined. Seeds can contain up to 50% oil, and they have been used for centuries in traditional medicine, against problems with the kidneys or the urinary tract, and against tapeworms [25,26].

With the close relationships between Se and I in the functioning of the thyroid gland and thyroid hormone biosynthesis and metabolism, we tried to find out if fortification with both of these elements at the same time is feasible for pumpkins. Our aims were to improve food nutritional quality and yield, and we investigated the following: (1) how plants take up added Se and I (e.g., in which form, possible interactions, etc.); and (2) the individual or combined effects of Se and I on growth, crop yield and the physiological and biochemical characteristics of the plants. 

## 2. Material and Methods

### 2.1. Growing Sprouts in Growth Chamber

The study of the pumpkin sprouts was performed in February 2014 in a growth chamber at the Biotechnical Faculty, University of Ljubljana (Slovenia). Pumpkin seeds (*Cucurbita pepo* L., cultivar ‘Slovenska Golica’) were soaked in 200 mL water (MilliQ; MerkMillipore, Burlington, MA, USA) or in aqueous solutions of Se at 10 mg/L and/or I at 1000 mg/L in the following combinations: selenate (SeO_4_^2−^ alone); selenite (SeO_3_^2−^ alone); iodide (I^−^ alone); iodate (IO_3_^−^ alone); SeO_4_^2−^ + I^−^; SeO_4_^2−^ + IO_3_^−^; SeO_3_^2−^ + I^−^; and SeO_3_^2−^ + IO_3_^−^. The dry seeds were weighed before and after the soaking treatments to determine the amount of water absorbed by the seeds. On average, after 6 h of soaking, 97.1 ±4.2 mg water was absorbed by each seed. On the assumption that Se and I were taken up by the seeds in the same way as water, this indicated that 0.971 µg Se (as SeO_4_^2−^ or SeO_3_^2−^) was absorbed per seed, and 97.1 µg I (as I^−^ or IO_3_^−^) was absorbed per seed. After the soaking, the seeds were rolled into filter paper, with 30 seeds per roll, and put into a glass, which was put into the growth chamber. The experiment was designed as two-factorial, in three randomised replicates. During germination, the seeds in the rolls were given tap water at 100 mL per roll. The pumpkin sprouts were grown in a growth chamber for 10 days from the end of February 2014 to the first week of March 2014, under controlled conditions with mean day/night temperatures of 22 °C/18 °C, relative air humidity from 65% to 85%, and 16 h of daylight. 

The sampling of the sprouts was performed when they were growing out of the rolls and their cotyledons were fully extended (at the age of cca. 10 days). Physiological measurements (i.e., photochemical efficiency of photosystem (PS)II, electron transport system (ETS) activity of mitochondria) were performed on fresh leaves 2 days before sampling. Later, the anthocyanin, photosynthetic pigments and tocopherol contents were determined in the leaves. To obtain the average sprout mass, all of the fully developed sprouts were taken as whole sprouts and were counted and weighed. The sprouts were than lyophilised at −50 °C (1-16 LSC; Christ Gamma), milled and homogenised with a planetary micro mill (MM 200; Retsch, Haan, Germany). The samples were stored at −20 °C until the analysis of their Se and I contents. 

### 2.2. Plant Treatments in the Field Experiment

The field experiment was carried out in an experimental field of the Biotechnical Faculty (University of Ljubljana, Slovenia; latitude, 46°2′ N; longitude, 14°28′ E; altitude, 298 m above sea level.). The soil of the experimental site was classified as gleic fluvisol. The 0–30-cm soil layer contained 26 g/kg soil organic matter, 22 mg/kg assimilable phosphorus and 26 mg/kg soil assimilable potassium. The mean initial soil nitrate content was 14 mg/kg, and the soil pH was 6.3. 

Pumpkin seeds (*Cucurbita pepo* L., cultivar ’Slovenska Golica’) were sown in April 2014 in polystyrene plug trays, and 1 month later the seedlings were transplanted to a raised bed (0.6 × 1.1 m). Each plot (1.8 × 1.1 m) contained 3 plants that were grown in one row in the middle of the bed. The experiment was laid out in a two factorial block design, with three randomised replicates. Three weeks after transplanting (i.e., at the flowering stage), the plant foliage was sprayed one time during the growth season, with 150 mL per plot of solution of the same concentrations and combinations of Se and I as was used for the seed soaking treatments (see above). The terminal ETS activities and the anthocyanin levels were measured for the leaves three times, at 10, 20 and 30 days after the Se and I treatments. Photosynthetic pigments and tocopherol contents were determined in leaves 20 days after the foliar treatments. At an interval 14 weeks after transplanting, when the majority of the pumpkin fruits had reached their ripe phase, the pumpkins were counted and weighed. The seeds from each pumpkin were removed, washed with water, dried and weighed, and then lyophilised (1-16 LSC; Christ Martin Christ Gefriertrocknungsanlagen GmbH, Osterode am Harz, Germany), milled in a knife mill (Grindmix GM 200; Retsch, Haan, Germany), and stored at −20 °C for further analysis. 

Data for the temperature and precipitation during the experimental period (May to September 2014) were obtained from the meteorological station on the experimental field of the Biotechnical Faculty (University of Ljubljana). During the study, the mean daily temperature ranged from 15.9 °C (May) to 20.8 °C (July), and the total precipitation was 764 mm, with the highest precipitation in September (203.6 mm) and the lowest in May (94 mm).

### 2.3. Selenium and Iodine Determination

For Se determination in the pumpkin sprouts, 0.20 g samples of lyophilised and milled sprouts were digested in 50 mL PTFE tubes with the following reagents: 96% H_2_SO_4_, 65% HNO_3_, 30% H_2_O_2_, 40% HF and 30% HCl. The samples were diluted with Milli-Q water. The Se contents were determined by hydride generation atomic fluorescence spectrometry (Excalibur; PS Analytical, Orpington, UK). The full procedure was as described by Smrkolj and Stibilj [27]. 

For Se determination in the pumpkin seeds, 0.25 g of lyophilised and milled pumpkin seeds were digested with 4 mL HNO_3_ (Merck) in a microwave oven (Ultrawave; Milestone, Shelton, CT, USA) with the following programme: 20 min ramp to 220 °C and 15 min hold at 220 °C. The post-digestion solutions were cooled to room temperature and diluted to the appropriate volume. The Se contents were measured using inductively coupled plasma–tandem mass spectrometry (ICP-MS, 7500ce Agilent Technologies, Tokyo, Japan). Accuracy and precision were checked with standard reference material: NIST 1567b (Wheat flour) and NIST 1570a (Spinach leaves). A good agreement between the determined (1175 ± 28 ng/g, 117 ± 22 ng/g, respectively) and certified (1140 ± 10 ng/g, 117 ± 8 ng/g, respectively) values was obtained. 

For I determination, 0.15 g samples of lyophilised milled pumpkin seeds or sprouts, 10 mL of water (Milli-Q) and 2 mL of 25% tetramethylammonium hydroxide were mixed in a glass vessel. Extraction of I was performed in a microwave oven, followed by determination of I content by inductively coupled plasma–tandem mass spectrometry. The detailed procedure was as reported previously [20]. The accuracy and precision of the data were checked using reference materials BCR 129 (Hay powder) and NIST SRM 1573a (Tomato leaves). The values obtained (±standard deviation) were 0.143 (±0.018) μg/g and 0.74 (±0.04) μg/g, respectively.

### 2.4. Biochemical and Physiological Analysis

The chloroplast pigments chlorophyll a, chlorophyll b, neoxanthin, violaxanthin, antheraxanthin, zeaxanthin, lutein, α-carotene and β-carotene were determined as previously described [28], and as detailed in Jerše et al. [20]. The method for the determination of the tocopherol concentrations, as α-tocopherol, γ-tocopherol and δ-tocopherol, was as reported by [28], with the detailed procedures described in [20,21]. 

The anthocyanin levels were determined according to Drumm and Mohr [29]. The absorbances of the extracts were measured at 530 nm using a UV/VIS spectrometer (Lambda 25; Perkin-Elmer, Norwalk, CT, USA). The anthocyanins were extracted from the weighed fresh plant material by homogenisation in a mortar and extraction with HCl: methanol (1:99; *v/v*). The absorbance of the extracts was measured at 530 nm using a UV/VIS spectrometer (Lambda 25; Perkin-Elmer, Norwalk, CT, USA). The anthocyanin levels are expressed in relative units.

The respiratory potential was measured as described by [30], following the detailed procedure given by [31]. In brief, samples from each of the Se and I treatments were homogenised. The ETS activities were measured as the rate of tetrazolium dye reduction, and then converted to oxygen equivalents, as described by Kenner and Ahmed [32].

### 2.5. Statistical Analysis

The experiments with the pumpkin sprouts and the field experiments with the pumpkin plants were treated as two-factorial experiments in three replications. The determination of Se and I, and all of the physiological and biochemical measurements, were replicated as three to five laboratory replicates and three analytical replicates. The data were analysed, using Statgraphics Centurion XVII (Statgraphics, Herndon, VA, USA) with one-way analysis of variance (ANOVA), for significant differences between the Se and I combinations. Differences were tested using Duncan tests, at a significance level of 0.05. 

## 3. Results and Discussion

### 3.1. Pumpkin in a Growth Chamber and in the Field

Growth chamber: Germination is one of the most critical stages in the plant life cycle, which starts with the uptake of water by dry seeds, and results in elongation of the seed embryo [33,34]. Many environmental factors can influence the germination processes and further sprout growth. 

For seeds soaked in Se and I that germinated to sprouts in a growth chamber, there were significant differences between the Se and I treatments of the seeds as regards germination rates (range, 33–74%) (Table 1). With the control germination at 50.0%, this was reduced by the treatments with SeO_4_^2−^ alone (33.3%) and with each of the I forms alone, namely I^−^ and IO_3_^−^ (45.6%, 48.3%, respectively), although not significantly. The combination of SeO_4_^2−^ and I^−^ or IO_3_^−^ led to significant improvements in the seed germination rates compared to the control and SeO_3_^2−^ and SeO_4_^2−^ alone, and to the I forms alone (I^−^, IO_3_^−^). In contrast, there were no significant effects on the seed germination rates in the treatments with SeO_3_^2−^ in combination with either I^−^ or IO_3_^−^. On the other hand, Germ et al. [22] used the same concentrations and combinations of Se and I in their treatments for growing buckwheat microgreens, and they only observed decreases in germination rates following treatments with SeO_3_^2−^ and IO_3_^−^ alone, and no stimulating effects of other treatments, including combinations of Se and I. In general, there were no significant differences between these Se and I treatments of the seeds here as regards sprout mass (range, 0.66–0.80 g). Contrary to these results, a significant decrease in sprout mass was recorded in our previous studies: for peas, sprout mass was reduced by all of the individual Se and I treatments used, except for SeO_3_^2−^, as well as for all the combinations of Se and I treatments [20]; and for buckwheat, significant decreases in sprout yields were seen only for all of the treatments with these Se and I forms alone [22]. Furthermore, spinach plants grown in a hydroponic system showed decreases in shoot biomass when treated with 10 and 50 µmol L^−1^ IO_3_^−^, alone or in combination with SeO_4_^2−^, which indicated an interaction between Se and I [17].

Field experiments: For the field experiment with the foliar application of Se and I to the plants, pumpkins yields and seed production were not affected by these treatments (Table 2). In line with the present results, no significant improvements in seed or other yield types were reported for different biofortification treatments with Se and/or I, such as the soil fertilisation of lettuce and carrots, fortified nutrient solutions with hydroponically grown lettuce and spinach [15,17,35], and, as in our previous study, pea plants [21]. On the other hand, Blasco et al. [36] reported that fertilisation with I^−^ significantly decreased shoot biomass and reduced leaf levels of nitrate, while IO_3_^−^ application improved all of the nitrogen parameters, including nitrogen-utilisation efficiency, and also increased the biomass of the edible parts of lettuce. 

### 3.2. Selenium and Iodine Content

Growth chamber: The Se and I contents of the sprouts grown from the soaked seeds were analysed. Here, the Se contents of the resulting pumpkin sprouts were significantly increased compared to the control with Se treatment, regardless of the Se form and combinations with I, although the SeO_4_^2−^ treatments were more effective (Table 1). Selenate (SeO_4_^2−^) is a highly soluble compound that is easily available to plants [37]. To the best of our knowledge, there is no data in the literature for Se content in pumpkin sprouts from enriched seeds. A comparison can instead be made using the studies of Cuderman et al. [38] and Germ et al. [22], where a higher uptake of SeO_4_^2−^ was seen for buckwheat sprouts soaked in solutions of 10 mg/L SeO_4_^2−^ or SeO_3_^2−^. A higher accumulation of Se applied as SeO_4_^2−^, compared to as SeO_3_^2−^, was recently also shown for pea sprouts [20] and spinach plants [39]. 

Compared to the control, the I content in these pumpkin sprouts was significantly increased if the seeds were soaked in the I solution (both forms). Accumulation of I was more efficient when I was added in its iodide form (I^−^), compared to iodate (IO_3_^−^). Similar results were reported for pea sprouts [20]. On the other hand, in buckwheat microgreens, the uptake of I was more efficient when the seeds were soaked in IO_3_^−^, as compared to I^−^ [22]. Lawson [40] showed that I^−^ is more readily available to plants in the solution of soilless culture systems, while under field conditions it is subject to cumulative losses greater than with IO^3−^. Blasco et al. [41] concluded that the most suitable addition rate for hydroponic cultivation of lettuce plants was 40 µM or less, in the form of I^−^, because the biomass was not reduced. Whitehead [42] and Zhu et al. [43] explained that it possible that IO^3−^ must be reduced to I^−^ before it can be taken up by plants, which is a process that requires energy. Thus, I accumulation in plants might be limited by the reduction process itself. 

Both forms of I in the soaking solutions significantly increased the sprouts’ Se contents, compared to the single treatments with SeO_3_^2−^ or SeO_4_^2−^. The highest Se content was seen for sprouts from seeds treated with the combination of both elements, regardless of the forms used in the soaking solutions. The synergistic effects of I on Se uptake have also been shown in lettuce [15], *Brasica juncea* [44] and pea plants [21], while antagonistic effects have been reported for carrot [18] and lettuce [19].

The I contents in the sprouts from the seeds treated with I^−^ in combination with SeO_4_^2−^ were higher, compared to sprouts treated with I^−^ alone. Furthermore, in the sprouts from seeds treated with IO_3_^−^ in combination with both of the forms of Se, the I levels were higher than in the sprouts from the seeds treated with IO_3_^−^ alone. The positive effects of Se on I uptake have been shown for *Brasica juncea* [44] and for pea sprouts [20]. On the other hand, Se hindered the accumulation of I in spinach (slightly) [17], carrot [18] and lettuce [19], and in the leaves of kohlrabi plants [16]. 

Both of these elements are transported from the roots to the vital organs through the xylem [45]. The presence of I amplified the Se uptake in these pumpkin seeds during their soaking. It is possible that pumpkin seeds have a permeable testa for Se and I, which can in turn accelerate the uptake of both elements. Iodine might have enhanced the permeability of the membrane to Se in order to provide this significant reinforcing effect. In the literature, controversial data on the effects of I on Se uptake, and vice versa, have been reported, depending on the form of the Se and I, and on crop type [16,18,20,21,22]. 

Field experiment: Seed Se contents following the foliar spraying of the pumpkin plants were slightly increased, compared to the control plants (Table 2). There were no significant differences in the Se contents of these pumpkin seeds between the leaves sprayed with SeO_3_^2−^ and SeO_4_^2−^. A study by Smrkolj et al. [24], also on pumpkin (*Cucurbuta pepo* L.) seeds, showed that foliar spraying of the pumpkin plants with natrium selenite at 1.5 mg/L resulted in eightfold increase in Se content compared to the untreated plants. The reasons for the low Se contents in our study might include (i) the small absorption of the foliar solutions, (ii) the higher yield of pumpkin seeds under the Se treatments, or (iii) the distribution of Se uptake throughout the pumpkin plants. The foliar applications of I^−^ and IO_3_^−^ did not result in enriched I contents in the pumpkin seeds. Indeed, I contents were even lower than for the control seeds. The reasons for these low I contents might include (i) the distribution over the mass of the fruit, developed between spraying and sampling (3.5 months), and (ii) the low I mobility through the phloem. 

The treatments with both I forms in combination with SeO_4_^2−^ increased the seed Se content up to 2.5-fold, compared to the control seeds. The highest Se contents were obtained when the pumpkin plants were sprayed with SeO_4_^2−^ + IO_3_^−^ (87 ng/g DW) and SeO_4_^2−^ + I^−^ (74 ng/g DW). These data show that I had a positive impact on the uptake of SeO_4_^2−^ from the leaf surfaces, and further Se translocation to the seeds. This is comparable with the SeO_4_^2−^ and IO_3_^−^ treatments in the case of lettuce, where a higher uptake of SeO_4_^2−^ was obtained in the presence of IO_3_^−^ [15]. In a study by Smoleń et al. [18], I in the forms of I^−^ and IO_3_^−^ decreased Se content when applied together with SeO_3_^2−^.

The presence of Se in the foliar solutions with I resulted in some significant differences in seed I contents across the treatments with I alone. For the plants treated with foliar spraying with SeO_3_^2−^ + I^−^ and SeO_4_^2−^ + IO_3_^−^, the seed I contents were comparable to the control seeds. On the other hand, I levels were lower in comparison to the control for all the other treatments. SeO_3_^2–^, but not SeO_4_^2−^, enhanced I levels when they were added in the form of I^−^. SeO_3_^2–^ did not enhance I levels when added in the form of IO_3_^−^, while SeO_4_^2−^ enhanced them. 

I acted synergistically on Se accumulation in sprouts and seeds, but the effects in sprouts were seen for all combinations, whereas in seeds they were lower, and only seen for SeO_4_^2−^. Although the patterns are repeated, the mechanisms of this correlation remain to be discovered, as different crops react differently according to the forms of Se and I used.

### 3.3. Biochemical and Physiological Characteristics of Sprouts and Plants

Measurement of the biochemical and physiological characteristics of the sprouts and plants showed that these Se and I treatments did not cause stress to the plants. There were no differences between the control and the Se and I treatments with regard to the levels of chlorophyll a, chlorophyll b, neoxanthin, violaxanthin, antheraxanthin, zeaxanthin, lutein, α-carotene, β-carotene and tocopherols, either in sprouts from the seed treatments or in the developed plants following the foliar treatments (data not shown). 

Growth chamber: However, there were some small differences among the treatments with regard to the anthocyanin contents. The anthocyanins in the sprouts from seeds soaked in different combinations of Se and I were similar to the control group. SeO_4_^2−^ in combination with I^−^ lowered the anthocyanin levels compared to the treatment with I^−^ alone, while both forms of Se in combination with IO_3_^−^ increased the anthocyanin levels compared to the treatment with IO_3_^−^ alone, in sprouts.

Field experiment: For the foliar treatments of the plants, there was minimal difference between the anthocyanin levels in the treated and untreated leaves, and no difference between the anthocyanin levels in the resulting seeds (Table 3). The anthocyanin levels also remained similar to those in the control plants of pea sprouts from seeds treated with the same combinations of Se and I as in the present study [20]. Some stressors can promote anthocyanin synthesis [46]. However, in the present study, the anthocyanin levels generally did not differ between the control and treated groups in sprouts, or between the leaves of mature plants and the seeds. The highest anthocyanin levels were seen for the foliar treatment of the leaves of mature plants. It should be noted that the light intensity is stronger in the field, and the plants can synthesise anthocyanins to protect their photosynthetic apparatus from photo damage [47,48].

Growth chamber: ETS activities in the sprouts from the Se and I-treated seeds were similar to the control for the majority of the treatments (Table 4). Enhanced ETS activity in the sprouts was observed for the treatments with SeO_3_^2−^ in combination with I^−^ and IO_3_^−^. Field experiment: ETS activity did not differ in the leaves from the control and treated plants following the foliar treatments. Spraying with both forms of Se enhanced ETS activity in the resulting seeds, in comparison to the controls, well as compared to all of the other treatments.

Field experiment: Similar ETS activities in seeds from the control and treated plants were seen in our previous study [21], wherein pea plants were treated with the same concentrations and combinations of Se and I. Considering these treatments with SeO_3_^2−^, SeO_4_^2−^, I^−^ and IO_3_^−^, there is scarce information available about the ETS activities in treated plants [16,20,21,22]. The effects of these treatments appear to be dependent on the crop, plant part and forms of Se and I. There was a trend in the declining of ETS activity, from the first to the third measurement, in the leaves of the control and treated plants (Table 3 and Table 4, Leaves 1–3). Plants require more energy during intensive growth and development, in order to build their structural components. Similar data were reported by Smrkolj et al. [49], where pea plants underwent foliar spraying with an aqueous solution containing Se 10 mg/L in the form of sodium selenate. 

The ETS activities were the same for sprouts and developed plants, while they were only about one tenth of these for seeds. This might be connected with the low oxygen supply in the pumpkin fruit [23]. Similarly, Borisjuk et al. [50] reported that respiration in the developing faba bean embryos (*Vicia faba*) is limited by hypoxia.

## 4. Conclusions

The pumpkin sprouts and seeds accumulated both Se and I. Both of these elements were translocated from the soaked seeds to the sprouts in the growth chamber experiment, and from the treated leaves to the seeds of plants in field experiment. Transportation from the leaves to the seeds was lower than translocation from the soaking solutions to the sprouts. The synergistic effects of I on Se uptake in the sprouts can be attributed to the effects of I on the permeability of the testa for Se. This mechanism needs to be studied in further detail. The results show that the application of Se and I can be an effective strategy for enhancing those elements in pumpkin plants, without negative impacts on the yield. The amounts of Se and I in 100 g of fresh pumpkin sprouts and seeds from the present study did not exceed the recommended daily intake of these two elements. However, in the supplementation of food with iodine and selenium caution is recommended, so as to diminish the risk of overdosing.

## Figures and Tables

**Table 1:** Effects of the various seed-soaking treatments on the characteristics of the pumpkin sprouts and their Se and I levels. Treatments were based on Se at 10 mg/L and I at 1000 mg/L.

Treatment	Germination (%)	Sprout Mass (g)	Sprout Selenium (µg/g DW)	Sprout Iodine (µg/g DW)
Control	50.0 ± 5.5 ^bc^	0.68 ±0.06	0.037 ± 0.005 ^f^	0.8 ± 0.1 ^e^
SeO_3_^2−^	57.8 ± 2.9 ^bc^	0.76 ± 0.04	0.197 ± 0.009 ^e^	
SeO_4_^2−^	33.3 ± 9.6 ^c^	0.70 ± 0.01	0.453 ± 0.013 ^d^	
I^−^	45.6 ± 6.8 ^bc^	0.74 ± 0.08		279 ± 19 ^b^
IO_3_^−^	48.3 ± 5.6 ^bc^	0.66 ± 0.08		217 ± 13 ^c^
SeO_3_^2−^ + I^−^	50.0 ± 5.5 ^bc^	0.77 ± 0.002	0.801 ± 0.079 ^c^	314 ± 1 ^ab^
SeO_3_^2−^+ IO_3_^−^	73.3 ± 17.8 ^ab^	0.74 ± 0.05	0.994 ± 0.06 ^b^	288 ± 25 ^ab^
SeO_4_^2−^ + I^−^	74.4 ± 14.4 ^a^	0.80 ± 0.04	2.0 ± 0.1 ^a^	323 ± 12 ^a^
SeO_4_^2−^ + IO_3_^−^	74.4 ± 18.9 ^a^	0.76 ± 0.04	2.3 ± 0.7 ^a^	266 ± 28 ^b^

Data are means ± standard deviation; ^a–f^ different superscript letters indicate statistically significant differences between the different treatments (*p* < 0.05).

**Table 2:** Effects of the various foliar treatments on the characteristics of pumpkin yields and seed Se and I levels. Treatments were based on Se at 10 mg/L and I at 1000 mg/L.

Treatment	Pumpkin Yield (t/ha)	Seed Yield (t/ha)	Seed Selenium (ng/g DW)	Seed Iodine (ng/g DW)
Control	92.7 ± 14.4	3.0 ± 0.7	35 ± 3 ^d^	30 ± 10 ^ab^
SeO_3_^2−^	115.5 ± 17.0	3.8 ± 0.9	51 ± 8 ^c^	
SeO_4_^2−^	114.9 ± 32.6	3.6 ± 2.1	44 ± 11 ^c^	
I^−^	60.7 ± 2.2	1.9 ± 0.1		9 ± 1 ^d^
IO_3_^−^	98.8 ± 11.0	3.4 ± 0.5		8 ± 2 ^d^
SeO_3_^2−^ + I^−^	61.7 ± 12.5	2.1 ± 0.5	46 ± 9 ^c^	31 ± 3 ^ab^
SeO_3_^2−^+ IO_3_^−^	89.5 ± 19.3	2.7 ± 0.7	43 ± 10 ^c^	15 ± 3 ^cd^
SeO_4_^2−^ + I^−^	102. 8 ± 16.8	2.9 ± 0.4	74 ± 8 ^b^	16 ± 1 ^cd^
SeO_4_^2−^ + IO_3_^−^	76.3 ± 4.7	2.3 ± 0.2	87 ± 5 ^a^	20 ± 4 ^bc^

Data are means ± standard deviation (*n* = 3); ^a–d^ different superscript letters indicate statistically significant differences between the different treatments (*p* < 0.05).

**Table 3:** Effects of the various seed and foliar treatments on the anthocyanin levels in the pumpkin sprouts, leaves of mature plants, and seeds.

Treatment	Anthocyanin Content (Rel. Units/g DW)				
	Sprouts	Leaves			Seeds
		1	2	3	
Control	77.1 ± 22.2 ^bc^	221.1 ± 18.2	383.0 ± 139.6 ^ab^	233.2 ± 24.2	131.2 ± 38.3
SeO_3_^2−^	79.2 ± 22.0 ^bc^	222.7 ± 16.1	440.6 ± 113.0 ^b^	254.5 ± 157.6	84.4 ± 26.4
SeO_4_^2−^	61.9 ± 11.6 ^b^	173.6 ± 35.8	267.5 ± 81.4 ^ab^	298.2 ± 69.7	114.2 ± 51.9
I^−^	100.6 ± 4.6 ^c^	221.8 ± 34.5	378.0 ± 166.4 ^ab^	278.8 ± 152.7	126.3 ± 63.8
IO_3_^−^	30.2 ± 20.8 ^a^	322.2 ± 6.2	403.8 ± 83.9 ^ab^	262.7 ± 59.0	132.8 ± 54.4
SeO_3_^2−^ + I^−^	86.9 ± 7.1 ^bc^	177.4 ± 54.7	311.6 ± 25.5 ^ab^	265.2 ± 134.6	102.7 ± 38.7
SeO_3_^2−^+ IO_3_^−^	72.7 ± 4.7 ^b^	206.4 ± 59.0	335.1 ± 95.3 ^ab^	163.7 ± 48.8	159.3 ± 73.6
SeO_4_^2−^ + I^−^	69.1 ± 2.3 ^b^	242.8 ± 30.2	235.9 ± 44.7 ^a^	141.9 ± 23.5	131.8 ± 32.6
SeO_4_^2−^ + IO_3_^−^	67.5 ± 12.6 ^b^	219.8 ± 10.3	261.8 ± 61.5 ^ab^	197.2 ± 68.7	128.0 ± 46.8

Data are means ± standard deviation (*n* = 3; leaves of mature plants, seeds), (*n* = 4; sprouts). Leaves 1, 2, 3, the three measurements of the leaves, at 10, 20 and 30 days after Se and I treatments. ^a–d^ different superscripted letters indicate statistically significant differences between the different treatments (*p* < 0.05).

**Table 4:** Effects of the various seed and foliar treatments on the activities of the electron transport system in the pumpkin sprouts, leaves of mature plants and seeds.

Treatment	Electron Transport System Activity (Rel. Units/g DW)				
	Sprouts	Leaves			Seeds
		1	2	3	
Control	9.82 ± 1.54 ^a^	14.99 ± 3.45	13.01 ± 0.94	8.27 ± 3.14	0.11 ± 0.08 ^ab^
SeO_3_^2−^	10.31 ± 0.40 ^ab^	21.33 ± 2.22	12.06 ± 3.81	8.14 ± 5.79	0.27 ± 0.20 ^c^
SeO_4_^2−^	11.01 ± 2.46 ^ab^	18.92 ± 3.85	14.87 ± 3.13	8.41 ± 3.16	0.19 ± 0.07b ^c^
I^−^	12.19 ± 1.74 ^abc^	17.00 ± 2.04	14.10 ± 3.33	10.00 ± 2.56	0.12 ± 0.04 ^a^
IO_3_^−^	11.19 ± 1.05 ^ab^	15.01 ± 4.12	10.79 ± 1.06	10.51 ± 4.04	0.10 ± 0.05 ^ab^
SeO_3_^2−^ + I^−^	12.43 ± 0.51 ^bc^	19.64 ± 0.63	11.12 ± 0.68	8.09 ± 0.84	0.16 ± 0.03 ^ab^
SeO_3_^2−^+ IO_3_^−^	14.42 ± 2.26 ^c^	20.29 ± 4.30	14.90 ± 3.23	7.39 ± 2.13	0.07 ± 0.04 ^a^
SeO_4_^2−^ + I^−^	11.0 ± 0.51 ^ab^	21.97 ± 4.38	12.35 ± 0.67	7.61 ± 1.64	0.15 ± 0.04 ^ab^
SeO_4_^2−^ + IO_3_^−^	11.58 ± 2.95 ^abc^	21.37 ± 5.40	13.70 ± 2.47	7.52 ± 1.69	0.10 ± 0.05 ^ab^

Data are means ± standard deviation (*n* = 3; leaves of mature plants, seeds), (*n* = 4; sprouts). Leaves 1, 2, 3, measurements of the leaves, at 10, 20 and 30 days after Se and I treatments. ^a–d^ different superscripted letters indicate statistically significant differences between the different treatments (*p* < 0.05).
